# Effects of Freeze–Thaw Cycles on the Internal Voids Structure of Asphalt Mixtures

**DOI:** 10.3390/ma15103560

**Published:** 2022-05-16

**Authors:** Di Yu, Haosen Jing, Jianan Liu

**Affiliations:** 1 School of Civil Engineering and Architecture, East China Jiaotong University, Nanchang 330013, China; yudi@ecjtu.edu.cn; 2 School of Materials Science and Engineering, Chang’an University, Xi’an 710061, China; 3 College of Transportation, Jilin University, Changchun 130025, China

**Keywords:** asphalt mixture, freeze–thaw cycle, X-ray computed tomography (CT), image processing, void, equivalent ellipse

## Abstract

Freeze–thaw cycle is one of the main distresses of asphalt pavement, and the law of freeze–thaw damage has always been an important topic. In this paper, X-ray computed tomography (CT) of asphalt mixture before and after freezing and thawing was carried out, and its two-dimensional (2D) digital image was recognized. Firstly, the eigenvalues of internal voids of asphalt mixture are extracted. Then the distribution of internal voids was analyzed. Finally, the evolution law of internal voids was summarized. The research results show that the characteristic mean value of the 9th cycle is the irreversible limit of freeze–thaw damage, and the non-resilience after the large void area increases is the fundamental reason for the accumulation of freeze–thaw damage. The source of void damage shifts from large voids to small voids, and the middle-stage is a critical stage of freeze–thaw damage. This work quantitatively evaluates the internal freeze–thaw damage process of asphalt mixture, and a morphological theory of the evolution of void damage based on an equivalent ellipse is proposed, which is helpful for better understanding the freezing–thawing damage law of asphalt pavement.

## 1. Introduction

Freeze–thaw cycle is one of the main distresses of asphalt pavement [1,2]. The icing on the road surface reduces the friction coefficient of the road and affects the driving safety of the vehicle. After melting, the water generates dynamic water pressure under the action of vehicle load, resulting in deepening of cracks and the peeling of aggregates [3]. The ice expands inside the pavement, resulting in material tension, which promotes the formation of crack networks and reduces mechanical properties [4]. After melting, water penetrates into the gap to deepen the damage.

In order to solve the freezing and thawing problem, a variety of methods have been proved to be effective. Snow-melting agent is widely used to melt ice on road surfaces, but excessive snow-melting agent will accelerate freeze–thaw damage [5]. Asphalt and aggregates with a better low temperature performance are a solution [6,7,8,9]. Fiber is a typical freeze–thaw resistant material, which can significantly improve the mechanical properties of asphalt mixture after freeze–thaw [10,11,12,13,14,15]. Besides the traditional material replacement and addition, some new methods are emerging. The properties of microwave heating steel slag were utilized to provide new possibilities for melting road surface ice and internal damage healing [16,17,18]. A good effect can be achieved by weakening the binding force of the ice layer with coatings [19]. Ice breaking capability was increased by road surface filled rubber elastomer [20]. While the improvement of freeze–thaw methods tends to diversify, the research on the mechanism of freeze–thaw cycles is also continuing to advance.

Traditional evaluation methods for freeze–thaw damage mainly rely on the degradation of macroscopic mechanical properties, such as freeze–thaw split ratio, split tensile strength, indirect tensile stiffness modulus, fatigue and creep, etc. [10,21,22,23,24]. Some scholars believe that the degree of freeze–thaw damage can be quantified by cracks. For example, fracture energy was quantified under different moisture freeze–thaw cycles [25]. However, the recent ASCE meeting shows that the cracking tolerance index was unable to provide the expected performance trends for asphalt mixtures subjected to a freeze–thaw cycle [24]. Because the mechanical properties of asphalt mixtures can only indirectly evaluate the effects of freeze–thaw damage, some scholars have tried to establish mechanical models to evaluate and predict freeze–thaw damage [26]. For example, based on the continuum damage theory, a freeze–thaw damage evolution model was established, and the predicted results agree well with the experimental data [27]. Finite element simulation was used with a variety of mechanical indicators, including fracture energy to evaluate the degree of freeze–thaw damage in combination [28]. In addition, adhesion was used to evaluate the degree of freeze–thaw damage by some scholars. The number of edge points were quantified to predict the adhesion performance of different aggregates to asphalt [29]. A combination of photoelectric colorimetry, contact angle method, and water immersion method was used to evaluate freeze–thaw adhesion [30]. Image methods were used to evaluate the effect of snowmelt salt on adhesion under freeze–thaw conditions [31]. Compared with mechanical indicators and modeling, image methods are more intuitive and clearer.

CT images were used to obtain changes in volumetric moisture content, saturation, and the number of voids, and these indicators were used to quantify the moisture distribution within the specimen [32]. Shuyin Wu et al. [33] used CT to scan the effect of snow-melting agents on void changes under freeze–thaw conditions and found that the size and number of voids increased as the number of freeze–thaw cycles increased. An analysis of 3D modeling through CT images, volume expansion found an increase in number and fusion of voids [34,35]. The higher the initial void ratio, the more obvious the void changes, and the void has a strong correlation with the strength index. CT images were used to find the large voids and small voids were negatively and positively correlated with tensile strength, respectively [36]. Based on the features of connected voids in CT 2D images, a damage model was established and found that connected voids contributed to the increase in the number of voids [27]. Information entropy was used to describe the freeze–thaw damage stage in CT images [37]. Huining Xu et al. [38] established the relationship between information entropy and void characteristics, and proposed a new method for freeze–thaw damage analysis.

With the diversification of freeze–thaw solutions, new progress has been made in freeze–thaw mechanisms and evaluation methods. The traditional method to evaluate freeze–thaw damage by mechanical indexes has certain limitations. Although the use of modeling methods such as finite element can further explore the freezing and thawing mechanism, the relationship between the model and the actual specimen is questionable. Drawing on other subfields such as self-healing or adhesiveness, only specific damage can be assessed. In comparison, the method of CT image is more intuitive, comprehensive, and practical. The void feature is typical for evaluating freeze–thaw damage. Although the law of voids under freeze–thaw conditions has been analyzed by some scholars, it is often limited to changes in the indicators themselves. The evaluation of freeze–thaw damage for void evolution lacks the establishment of a theoretical system. Therefore, based on the image processing of CT 2D images, this paper extracts void features to achieve the following objectives. The distribution and morphological evolution of voids were quantitatively evaluated based on void characteristics; the theory of freeze–thaw damage is established according to the evolution law of void characteristics.

## 2. Materials and Methods

### 2.1. Materials

The properties of asphalt used in this work are shown in Table 1. All properties meet the requirements of the Technical Specification for Highway Asphalt Pavement Construction [39].

Properties of aggregates tested according to *Testing Procedures of Aggregate for Highway Engineering in China (JTG E42-2005)* [40] are shown in Table 2.

### 2.2. Asphalt Mixtures Design

**Figure 1:**
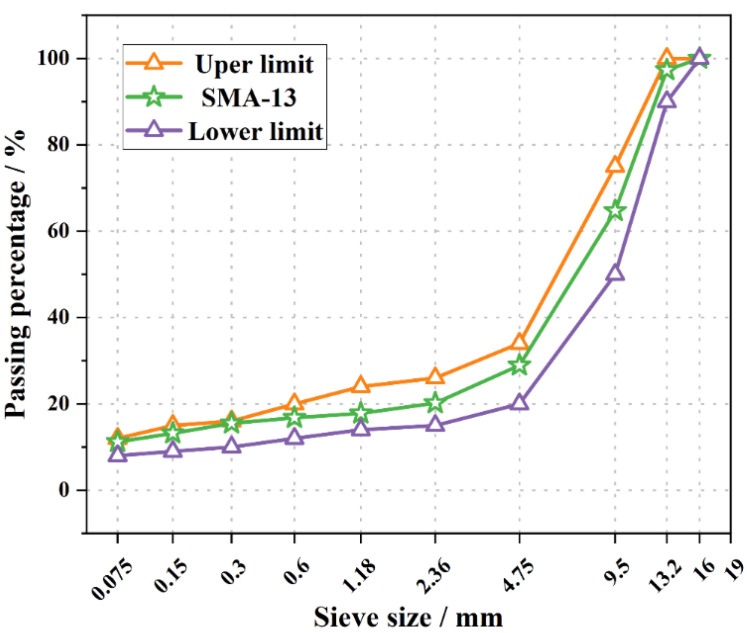
Gradation of asphalt mixtures.

The gradation of asphalt mixtures in this work is shown in Figure 1. The Marshall test was used to determine the optimal asphalt–aggregate ratio as 6.2% of SMA-13 mixtures.

### 2.3. Freeze–Thaw Cycle Test

The freeze–thaw cycle test was carried out on the asphalt mixture. Before scanning, the specimen was saturated with water under vacuum for 15 min, and then soaked under normal pressure for 30 min. The test pieces were first put into plastic bags; each bag was added to 10 mL of water and then placed in a low temperature box of −18 °C for 16 h, then placed in a constant temperature water tank at 60 °C to dissolve for 8 h as a freeze–thaw cycle. The number of freeze–thaw cycles was 0, 3, 6, 9, 12, and 15, and the samples were taken out and placed in an indoor environment of 20 °C for 7 d.

### 2.4. Image Processing

#### 2.4.1. CT Scan

**Figure 2:**
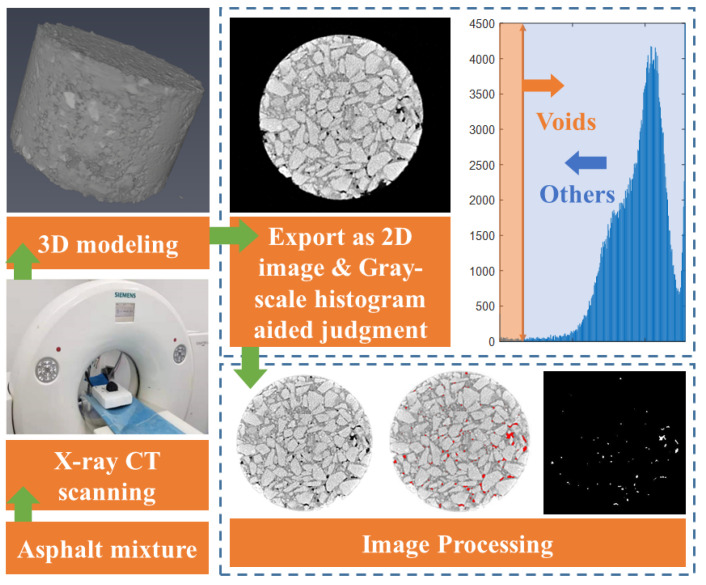
CT scanning and image processing.

The scanned CT images were obtained from the same Marshall specimen under 0, 1, 3, 6, 9, 12, and 15 times of freezing and thawing. As shown in Figure 2, after 3D modeling, a total of 1715 images were exported in BMP format, which contained incomplete images that were removed.

#### 2.4.2. Image Processing

The 60 complete images were randomly selected from the middle, with a total of 420 images in 7 groups as samples for analysis and processing. Due to certain differences in image quality, the number of voids extracted by different groups was quite different. In order to further control the number of samples and ensure the rationality of the analysis results, the area of each group of voids was sorted in descending order; the first 30 voids with the larger area of each image were taken, and the sample size of each group was controlled within 1544~1800 void points, with a total of 12,243 void points.

## 3. Results and Discussion

### 3.1. Void Distribution Law

#### 3.1.1. Void Feature

For better quantitative analysis, the void feature parameters used in this work are as follows:

(1) $$a_{mean}=(\sum_{i=1}^{k}a_{i})/k$$

(2) $$a_{\max}=\max\{a_{i}\}$$

(3) $$a_{gross}=\sum_{i=1}^{k}a_{i}$$

(4) $$d_{mean}=({\sum_{i=1}^{k}d}_{i})/k$$

(5) $$A_{mean}=(\sum_{j=1}^{n}a_{mean})/n$$

(6) $$D=(\sum_{j=1}^{n}d_{mean})/n,$$

where *n* is the number of image samples in each group (*n* = 60); *k* is the number of voids for each image (*k* ≤ 30); $a_{i}$ is the area of void and $d_{i}$ is the maximum diameter of the void.

#### 3.1.2. Changes in Void Characteristics

**Figure 3:**
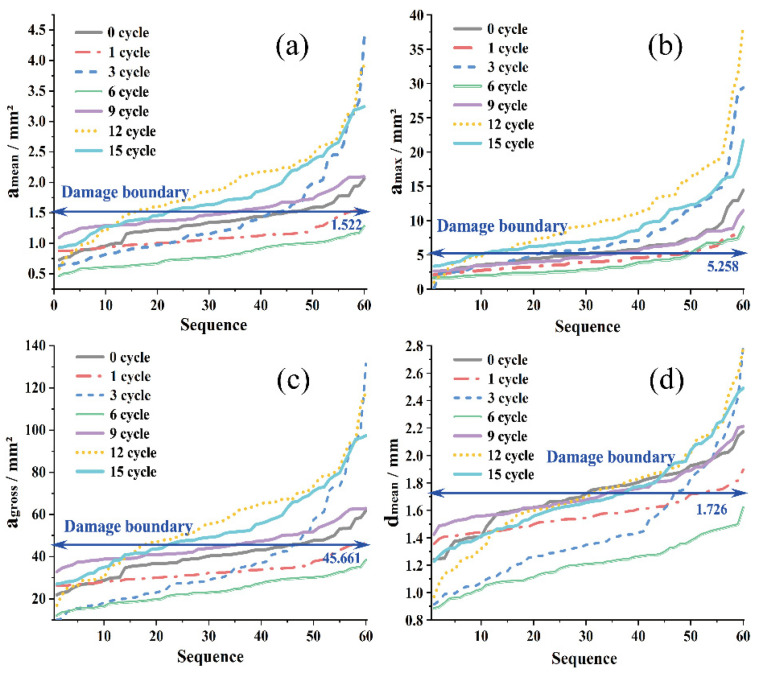
The change of indicators under different freeze–thaw cycles: (**a**) $a_{mean}$; (**b**) $a_{\max}$; (**c**) $a_{gross}$; (**d**) $d_{mean}$.

Figure 3 shows the change of different indicators under different freeze–thaw cycles. From Figure 3a, it can be seen that the average void area ($a_{mean}$) fluctuates with an increasing number of freeze–thaw cycles. However, under the action of 1~6 freeze–thaw cycles, the $a_{mean}$ fluctuated and decreased, and a significant overall increase did not appear until the ninth freeze–thaw cycle. Taking the feature mean of the ninth cycle as the damage boundary, it can be seen that most values of the area indicator exceed the boundary value at the 12th and 15th freeze–thaw cycles (Figure 3a–c), the overall durability drops by a gradient. The boundary discrimination of the average maximum diameter ($d_{mean}$) is not very clear (Figure 3d).

The 12th to 15th times of freezing and thawing after exceeding the damage boundary will increase the degree of irreversible damage caused by freezing and thawing, the rebound of large voids will decrease significantly, and the smaller voids will continue to fluctuate. It shows that the accumulation of irreversible damage may be more reflected in the “self-healing failure” of large voids (that is, no longer rebounding to a smaller value). The small voids continue to grow into large voids, and no longer being self-healing and the recovery may be the root causes of repeated freeze–thaw iterations [41]. In order to further understand this law, the probability distribution of the $a_{mean}$ was explored.

#### 3.1.3. Void Variation Distribution

**Figure 4:**
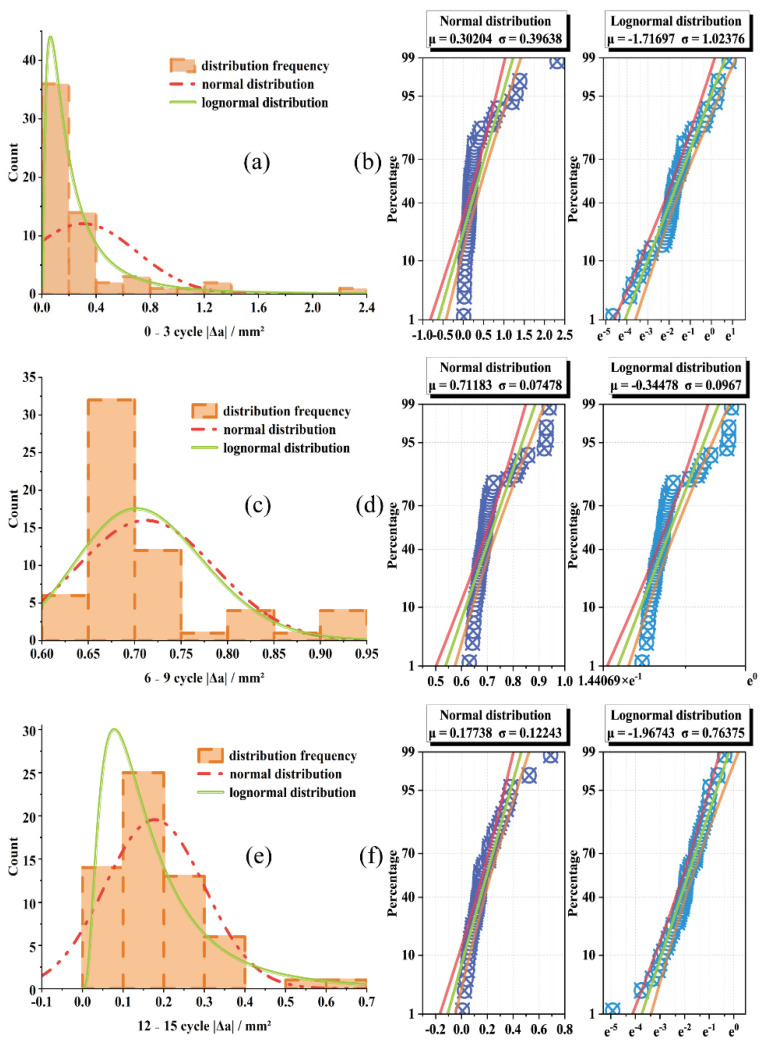
Frequency distribution histogram and probability plot. (**a**) 0~3 cycle distribution, (**b**) 0~3 cycle probability plot, (**c**) 6~9 cycle distribution, (**d**) 6~9 cycle probability plot, (**e**) 12~15 cycle distribution, (**f**) 12~15 cycle probability plot.

As shown in Figure 4, the freeze–thaw damage was divided into three stages: early stage, middle stage and late stage, corresponding to the changes of the void area |△a| in 0~3, 6~9 and 12~15 groups. We performed normal distribution and lognormal fitting, and found that the normal distribution function will cover the negative part. Comparing the probability plots of the two fitting types, it is found that the log-normal fitting is better and more in line with the actual distribution.

The statistical results of |△a| are shown in Table 3. From the mean value, it can be seen that the area of 6~9 cycles increased the most significantly, and the area of 12~15 cycles changed the least, indicating that the freeze–thaw damage mainly increased rapidly in the mid-term, and the damage slowed down after exceeding the damage boundary. According to the range and the coefficient of variation, the volatility of 0~3 cycles is the strongest, and the volatility of 6~9 cycles is the smallest. It shows that freeze–thaw damage mainly occurs in the collective expansion of voids in the mid-term, the early frost resistance of asphalt mixture is the strongest, and the source of void damage is transferred from large voids to small voids.

**Figure 5:**
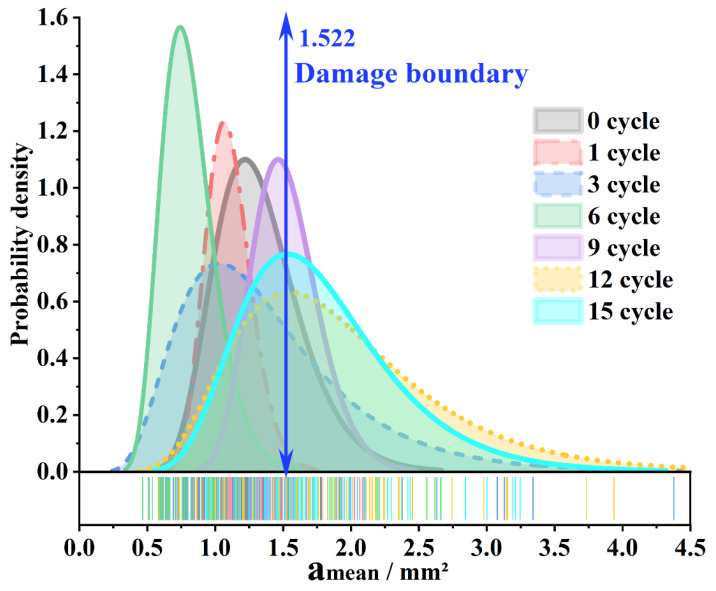
Probability density distribution.

Figure 5 shows the probability density plot of the $a_{mean}$, and it can be seen that the center of the probability density distribution fluctuates. However, by the ninth freeze–thaw cycle, there was an obvious right shift, the overall gap became larger, and then it was completely flat and the distribution range was wider. It fluctuated on a broad premise, and there was no apparent concentration again.

**Figure 6:**
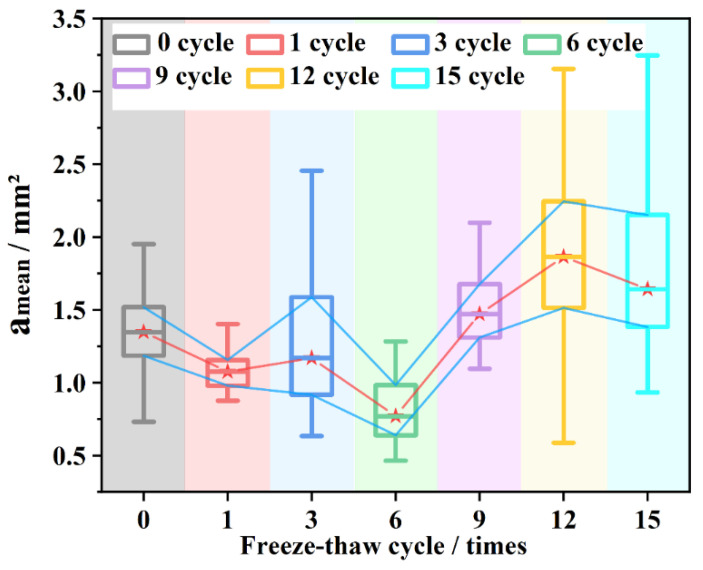
Distribution changes.

As shown in Figure 6, the median value of the average void area shows a fluctuating increase, and the distribution range also shows a fluctuating increase and then stabilizes. The 25% and 75% also show the same pattern, but the maximum value tends to stabilize after the volatility increases. The minimum value fluctuates greatly near the ninth freeze–thaw, indicating that the growth of voids may have a certain upper limit, and the maximum value tends to be stable after increasing to a certain range. The overall failure beyond the damage limit still originates from the collective growth of the smaller void area.

### 3.2. Distribution Quantification

#### 3.2.1. Kurtosis and Skewness

**Figure 7:**
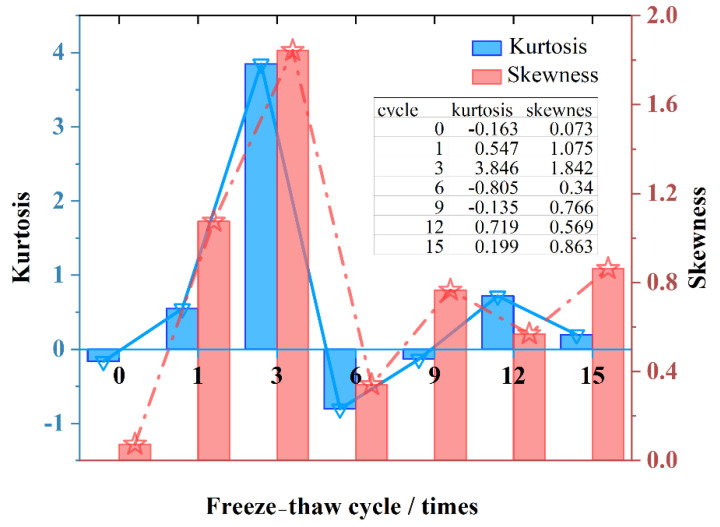
Kurtosis and skewness.

As shown in Figure 7, the kurtosis and skewness of the distribution curve (calculated according to Equations (7) and (8)) are analyzed. In statistics, kurtosis measures the kurtosis of the probability distribution of a real random variable. High kurtosis means that the increased variance is caused by low frequency extremes of a difference greater than or less than the mean. Skewness is a measure of the direction and degree of skewness in the distribution of statistical data, and a numerical characteristic of the degree of asymmetry in the distribution of statistical data. During 0~1 freeze–thaw times, the kurtosis continued to increase, indicating that the decrease in the $a_{mean}$ during the 0~3 freeze–thaw times in the early stage was mainly caused by the reduction of large voids. At this stage, the skewness is also increasing, indicating that the right skewness of the curve continues to increase, and the small voids and large voids show opposite characteristics. The small voids kept increasing in the early stage, but the area value fluctuated more when the large voids became smaller, reflecting the decreasing trend of the $a_{mean}$.

(7) $$K=\frac{n(n+1)}{\left(n-1)(n-2)(n-3\right)}{\sum_{i=1}^{n}\left(\frac{x_{i}-\bar{x}}{S_{a}}\right)}^{4}-\frac{3{\left(n-1\right)}^{2}}{\left(n-2)(n-3\right)}$$

(8) $$SK=\frac{\frac{1}{n}\sum_{i=1}^{n}{\left(x_{i}-\bar{x}\right)}^{3}}{(\frac{1}{n}\sum_{i=1}^{n}{\left(x_{i}-\bar{x}\right)}^{2}{)}^{3/2}},$$

where K is kurtosis; SK is skewness; n is the number of samples; $x_{i}$ is the $a_{mean}$ of ith void; $\bar{x}$ is $A_{mean}$; and $S_{a}$ is the variance of $a_{mean}$.

After the 1~3 cycles, the kurtosis and skewness continued to increase, reaching their respective peaks. $a_{mean}$ and $a_{\max}$ both increased (Figure 3), indicating that both large and small voids were getting larger. Larger voids account for a higher proportion of the overall change, indicating that the growth of smaller voids is not obvious enough, and there is no local sudden growth. Smaller voids collectively increase slightly, while large voids shrink.

After the sixth freeze–thaw, the kurtosis decreased significantly, even lower than the 0 value, and the skewness also decreased. It shows that the large voids shrink greatly, and the small voids also shrink. This shrinkage was non-uniform, resulting in a wider probability distribution and a larger decrease in mean-to-mean void area.

After the ninth freeze–thaw, the kurtosis and skewness increased again. At this time, the kurtosis was still less than 0, and the skewness increased again. Compared to kurtosis and skewness without freeze–thaw, the effect of large voids is flat. However, at this time, the maximum void area has become larger, and the overall curve is skewed to the right. It shows that the small voids also increased collectively during the freezing and thawing process, and the phenomenon of flattening appeared. It shows that under freeze–thaw damage, the generation of irreversible loss is not completely determined by large voids. Some of the smaller voids themselves have completed collective development, and at the same time, gradient transitions have been realized, and fluctuations around larger values are the main cause of irreversible damage reason.

After the 12th freeze–thaw, the kurtosis increased and the skewness decreased. At this time, the large voids increased again and, at the same time, the influence accounted for a larger proportion. The smaller voids also increased, and the overall average area increased.

After the 15th freeze–thaw, the kurtosis decreased, the skewness increased, and the area of large voids decreased. At the same time, the curve shifted to the right, indicating that the smaller voids increased again, and the distribution range increased slightly.

In general, the damage of freeze–thaw cycles has an effect on large voids, but is essentially caused by the collective enlargement of smaller voids. The collective development of small voids leads to an overall decrease in durability after reaching a certain level, resulting in permanent and irreversible damage.

#### 3.2.2. Freeze–Thaw Void Shape

After clarifying the overall change of voids under the increase of freezing and thawing times, it is necessary to further explore the form and shape of void growth. Comparing the $a_{mean}$ in Figure 3a with the $a_{\max}$ in Figure 3b, under the same freezing and thawing conditions, the change trend of the maximum void and the average void is basically the same during the first to sixth freeze–thaw processes. It shows that the freezing and thawing of the largest void and the smaller void are consistent in the early and middle stages. However, after the ninth freeze–thaw, the overall lift of the average void area curve was entirely derived from smaller voids, after which the maximum void area and the $a_{mean}$ fluctuated and increased again, with overall consistency.

Comparing (Figure 3a) $a_{mean}$ and (Figure 3d) average void maximum diameter change, it can be found that the $d_{mean}$ was basically unchanged after the ninth freeze–thaw cycle. At the same time, $d_{mean}$ is still increasing, indicating that the increase in void area after the overall failure is the widening of the short-axis direction, rather than the crack extension in the long-axis direction.

**Figure 8:**
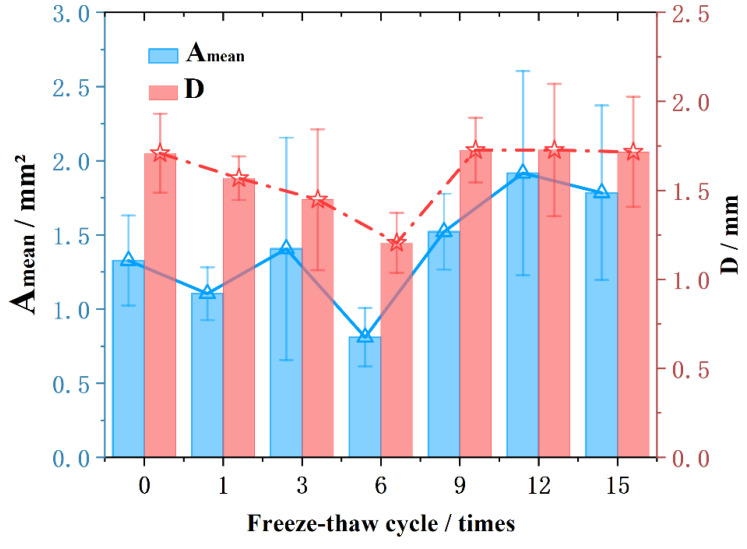
$A_{mean}$ and D under different freeze–thaw cycles.

As shown in Figure 8, both the $A_{mean}$ and D decreased after one freeze–thaw. After the third freeze–thaw, the D still decreased, but the $A_{mean}$ increased. It shows that the short-axis direction has increased, and the fluctuation of the originally stable area increase and decrease trend mainly comes from the expansion and contraction of the short-axis direction of the void. After the sixth freeze–thaw, the D decreased again, and $A_{mean}$ also decreased.

After the ninth freeze–thaw cycle, the D tended to be consistent. However, the $A_{mean}$ is still fluctuating and growing, and the D remains unchanged when increasing and decreasing. It shows that after the overall failure, the expansion and contraction of large voids all occur in the direction of the short axis, regardless of the direction of the long axis. The development of macrovoids after overall damage is determined by the shrinkage and expansion in the short axis direction.

In general, at the initial stage of freezing and thawing, the D continues to decrease, the short axis direction fluctuates and expands, and the shape of the pores is also closer to a sphere. After the void generally shrinks to a “sphere-like” threshold, the damage caused by re-freezing and thawing will quickly exceed the damage limit, resulting in the instability of the void structure. The maximum diameter and length tend to be stable after recovery, and continue to shrink and expand in the direction of the short axis.

### 3.3. Freeze–Thaw Void Morphology Theory

**Figure 9:**
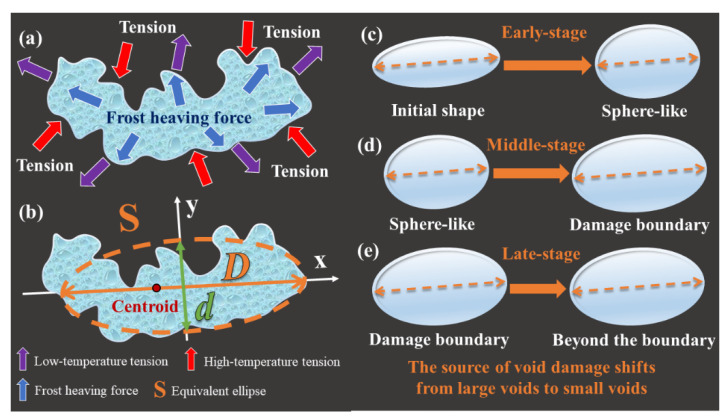
Theoretical diagram of freeze-thaw void morphology. (**a**) tensions schematic; (**b**) equivalent ellipse schematic; (**c**) early-stage morphological changes; (**d**) middle-stage morphological changes; (**e**) late-stage morphological changes.

According to the data analysis results, as shown in Figure 9, the theory of the evolution of freeze–thaw void morphology is proposed. The equivalent ellipse [42] S is obtained by using the maximum diameter D of the void. As shown in Figure 9c, in the early-stage of freezing and thawing, D decreases and the void area decreases. The fluctuation of d is the strongest, and the frost resistance of the asphalt mixture is stronger. In the middle-stage, as shown in Figure 9d, D grows rapidly, d fluctuates less, the void area increases collectively, and the durability decreases. In the late-stage of freezing and thawing, as shown in Figure 9e, D no longer shrinks, the fluctuation of d is small, and the source of freeze–thaw damage transfers from large voids to small voids. The processes of c→d→e are repeated, and new voids continually exceed the damage boundary, leading to damage iterations.

## 4. Conclusions

This work explores the distribution and variation of voids in SMA-13 asphalt mixture under different freeze–thaw cycles, and the conclusions are as follows.

1. The voids were irreversibly damaged after the ninth freeze–thaw cycle. The average values of the indicators of the ninth cycle are used as the damage boundary. After the damage boundary is exceeded, the degree of irreversible damage caused by freezing and thawing increases, the rebound of large voids decreases significantly, and the small voids continue to fluctuate. The accumulation of freeze–thaw damage is reflected in the springback failure of large voids;
2. From the statistical value of |Δa|, it can be seen that middle-stage (6~9 cycles) is the critical stage of freeze–thaw damage. The large void area tends to stabilize in the late-stage (12~15 cycles). The frost resistance of the asphalt mixture is the strongest in the early stage (0~3 cycles). The source of void damage shifts from large voids to small voids;
3. The fluctuation of void growth mainly comes from the contraction and expansion in the short axis direction. The main reason for the irreversible damage is the springback failure in the long axis direction, which leads to the collective increase of the small void area in 6~9 cycles. In the late-stage of freeze–thaw damage (12~15 cycles), the $d_{mean}$ remained stable, the source of damage shifted to small voids, and the fluctuations of damage and deepened short-axis fluctuations came from smaller voids.
4. According to the change trend of D, the morphological evolution of freeze–thaw voids is divided into three stages. In the early-stage, D shrinks, the shape of the void tends to be round, and the freeze–thaw resistance is strong. In the middle-stage, D grows, the void area grows steadily, and the durability decreases as a whole. In the late-stage, D remained stable, the void area fluctuated slightly but tended to be stable, and the freeze–thaw damage was irreversible and further deepened.

## Figures and Tables

**Table 1:** Properties of SBS asphalt.

Properties	Test Results	Test Methods (JTG E20-2011)
Penetration (25 °C, 100 g, 5 s; 0.1 mm)	65	T0604
Ductility (5 °C, 5 cm/min; cm)	43	T0605
Softening point (°C)	64	T0606

**Table 2:** Properties of aggregates.

Sieve (mm)	Apparent Specific Gravity (g/cm^3^)	Crushing Value (%)	Los Angeles Abrasion (%)	Water Absorption (%)
13.2~16	2.806	15.3	21.3	0.62
9.5~13.2	2.805	13.6	19	0.60
4.75~9.5	2.805	13.9	19	0.28
2.36~4.75	2.726	16.7	15.8	0.70
1.18~2.36	2.783	-	-	0.65
0.6~1.18	2.785	-	-	
0.3~0.6	2.765	-	-	
0.15~0.3	2.759	-	-	
0.075~0.15	2.716	-	-	

**Table 3:** Statistical results.

Cycle/Times	Mean Value	Range	Standard Deviation	Coefficient of Variation
0~3	0.302	2.304	0.396	1.312
6~9	0.712	0.309	0.075	0.105
12~15	0.177	0.683	0.122	0.690

## Data Availability

All data is contained within the article.
